# Marula oil nanoemulsion improves motor function in experimental parkinsonism *via* mitigation of inflammation and oxidative stress

**DOI:** 10.3389/fphar.2023.1293306

**Published:** 2023-11-23

**Authors:** Reem Alshaman, Mona Qushawy, Hatem I. Mokhtar, Angie M. Ameen, Rehab M. El-Sayed, Eman Saad Alamri, Lamiaa M. Elabbasy, Ahmed M. N. Helaly, Walid F. Elkhatib, Eidah M. Alyahya, Sawsan A. Zaitone

**Affiliations:** ^1^ Department of Pharmacology and Toxicology, Faculty of Pharmacy, University of Tabuk, Tabuk, Saudi Arabia; ^2^ Department of Pharmaceutics, Faculty of Pharmacy, University of Tabuk, Tabuk, Saudi Arabia; ^3^ Department of Pharmaceutics, Faculty of Pharmacy, Sinai University, El Arish, Egypt; ^4^ Department of Pharmaceutical Chemistry, Faculty of Pharmacy, Sinai University-Kantara Branch, Ismailia, Egypt; ^5^ Department of Physiology, Faculty of Medicine, Suez Canal University, Ismailia, Egypt; ^6^ Department of Pharmacology & Toxicology, Faculty of Pharmacy, Sinai University, El Arish, Egypt; ^7^ Food Science and Nutrition Department, Faculty of Science, University of Tabuk, Tabuk, Saudi Arabia; ^8^ Department of Medical Biochemistry and Molecular Biotechnology, Faculty of Medicine, Mansoura University, Mansoura, Egypt; ^9^ Department of Basic Medical Sciences, College of Medicine, Almaarefa University, Riyadh, Saudi Arabia; ^10^ Department of Forensic Medicine and Toxicology, Faculty of Medicine, Mansoura University, Mansoura, Egypt; ^11^ Microbiology and Immunology Department, Faculty of Pharmacy, Ain Shams University, African Union Organization St., Cairo, Egypt; ^12^ Department of Microbiology and Immunology, Faculty of Pharmacy, Galala University, Suez, Egypt; ^13^ Pharm D Program, Faculty of Pharmacy, University of Tabuk, Tabuk, Saudi Arabia; ^14^ Department of Pharmacology and Toxicology, Faculty of Pharmacy, Suez Canal University, Ismailia, Egypt

**Keywords:** experimental parkinsonism, inflammation, motor function, marula oil, oxidative stress

## Abstract

**Introduction:** Parkinson’s disease (PD) is a neurologic condition exhibiting motor dysfunction that affects old people. Marula oil (M-Oil) has been used longley in cosmetics and curing skin disorders. M-Oil is particularly stable due to its high concentration of monounsaturated fatty acids and natural antioxidants. The current study formulated M-Oil in an o/w nanoemulsion (M-NE) preparations and tested its anti-inflammatory and antioxidant actions against experimental parkinsonism.

**Methods:** Four experimental groups of male albino mice were used and assigned as vehicle, PD, PD + M-Oil and PD + M-NE. Locomotor function was evaluated using the open field test and the cylinder test. Striatal samples were used to measure inflammatory and oxidative stress markers.

**Results:** The results indicated poor motor performance of the mice in PD control group then, improvements were recorded after treatment with crude M-Oil or M-NE. In addition, we found high expression and protein of inflammatory markers and malondialdehyde levels in PD group which were downregulated by using doses of crude M-Oil or M-NE. Hence, formulating M-Oil in form of M-NE enhanced its physical characters.

**Discussion:** This finding was supported by enhanced biological activity of M-NE as anti-inflammatory and antioxidant agent that resulted in downregulation of the inflammatory burden and alleviation of locomotor dysfunction in experimental PD in mice.

## 1 Introduction

The plant *Sclerocarya birrea* (A. Rich.) Hochst (Marula), a member of the Anacardiaceae family, is a crucial tree for a number of African cultures (Deyama et al., 2001). One previous study has reported on its fatty acid content, such as oleic (C18:1), palmitic (C16:0), stearic (C18:0), and linoleic (C18:2) acids (Zharare and Dhlamini, 2000; Kleiman et al., 2008; Vermaak et al., 2011). Another previous study has reported on the antidiabetic potential of marula and suggested that it could prevent neurological complications of diabetes (Onaolapo and Onaolapo, 2020). Marula oil (M-Oil) has been used for moisturizing, maintaining healthy skin, and as an anti-aging agent (Deyama et al., 2001; Komane et al., 2015; Shoko et al., 2018). It has also long been used in cosmetics, in cooking oil, and to cure skin disorders (Mashau et al., 2022). M-Oil is particularly stable due to its high concentration of monounsaturated fatty acids and natural antioxidants (Mariod and Abdelwahab, 2012), to which its powerful antioxidant effects are attributed. Although its fatty acid makeup is similar to that of olive oil, M-Oil is ten times more resistant to oxidation (Wynberg et al., 2003; Mariod et al., 2008).

Nanoemulsions involve the dispersion of two immiscible liquids stabilized with a surfactant (Ahire and Gorle, 2021). They are divided into two varieties based on the proportion and chemical nature of the metabolites: water-in-oil and oil-in-water (Demisli et al., 2020). Nanoemulsions are prepared using either high- or low-energy emulsification processes (Gupta et al., 2019). The average droplet diameter attained is less than 500 nm (Bhatt and Madhav, 2011). Because of their small droplet size, they have a clear or hazy appearance, as opposed to the milky white color associated with coarse emulsions (Singh et al., 2017). Nanoemulsions can be delivered *via* a variety of routes, including topical, oral, intravenous, intranasal, pulmonary, and ophthalmic routes (Garcia et al., 2022), and have been reported to be effective in enhancing the biological activities of natural oils (Qushawy et al., 2022) or molecules (Bendary et al., 2020).

Parkinson’s disease (PD) is a neurological condition with the hallmark symptoms of rigidity, resting tremor, bradykinesia, and postural instability; it primarily affects the elderly (Rai et al., 2017; Rai et al., 2020; Alexander, 2022). The pathophysiology of PD is multifaceted, with many interacting processes and a complicated interplay of genetic and environmental factors. Numerous strands of evidence suggest that neuroinflammation is a causative factor in the neurodegeneration of these dopaminergic neurons (Teema et al., 2016). More recently, pharmacological manipulation using anti-inflammatory and antioxidant molecules has received a lot of attention for its potential to reduce neuronal damage (Buendia et al., 2016).

Damage to macromolecular components, such as DNA, proteins, and lipids, caused by oxidative stress (OS) leads to a variety of pathological states, such as PD (Gilgun-Sherki et al., 2001; Mythri et al., 2011). Decreased levels of antioxidant enzyme activity and lower levels of reduced glutathione (GSH) have been observed in numerous post-mortem investigations of PD patients’ brains (Blum et al., 2001; Blesa et al., 2015). Animal models of PD utilizing neurotoxins, including rotenone, corroborate the presence of OS in PD (Motawi et al., 2022). Antioxidant defense mechanisms evolve in response to OS to protect cells from injury by keeping redox equilibrium in balance (Trachootham et al., 2008).

For the overwhelming majority of PD patients, the disease’s pathogenesis and causes remain a mystery, despite extensive research into the mechanisms responsible for PD (Przedborski, 2017). All the current therapeutic approaches merely alleviate the motor symptoms, and there are not yet any curative or preventative medications that can halt the neurodegenerative deterioration. The current study aimed to examine formulations of M-Oil in o/w nanoemulsion (M-NE) preparations, to characterize these nanopreparations, and to identify the best formula to be tested as an anti-inflammatory and protective remedy against experimental parkinsonism in mice. The protective effect was examined in terms of reductions in the inflammatory burden and improvements in locomotor activity.

## 2 Materials and methods

### 2.1 The marula oil sample

The M-Oil sample applied in this study was a commercial M-Oil^®^ product from Pure Body Naturals, purebodynaturals.com (Cincinnati, Ohio, USA), purchased online through iHerb (iherb.com, Irvine, California, USA). The sample lot number was #21194, with a “best by” date of 13th July 2023. The M-Oil was provided in amber glass bottles containing 30 mL of 100% refined natural oil with a nutty aroma profile, obtained from wild-harvested and cold-pressed marula tree (*Sclerocarya birrea*) kernels cultivated in South Africa.

### 2.2 Oil sample characterization in terms of saponifiable and unsaponifiable content

Oil sample characterization consisted of two aspects: the profiling of saponifiable matter fatty acids and the profiling of the unsaponifiable matter metabolite in the provided oil sample, in accordance with the ConPhyMP checklist of items for conducting and reporting of analytical methods (Heinrich et al., 2020; Heinrich et al., 2022). The profiling of both saponifiable and unsaponifiable matter was accomplished by GC-MS determination, followed by comparison with the NIST11 standard library of MS fragmentation profiles of the acquired metabolites. The preparation of samples of the saponifiable and unsaponifiable matter in pure M-Oil followed previously published procedures (Qushawy et al., 2022), with some modifications. We took a sample of M-Oil (2 g), mixed it with a 15-mL sample of 10% ethanolic potassium hydroxide, and refluxed this for 5 h at a temperature of approximately 90°C–95°C to saponify the sample triglycerides. We allowed the refluxed oil sample to cool and then mixed it with 20 mL of distilled water. Next, we moved the diluted sample quantitatively into a 250-mL separating funnel and extracted it using 40 mL of petroleum ether. We collected the lower aqueous phase for preparation of the saponifiable matter transesterification.

For gas chromatography (GC) testing, we prepared the unsaponifiable matter sample by collecting the etherical phase. The ether layer was washed thrice with equal volumes of distilled water, then mixed with anhydrous calcium chloride to remove residual water, and filtered. The ether filtrate was dried at a temperature not exceeding 50°C until complete evaporation of the organic solvent. The weight of the collected residue was recorded.

For preparation of the saponifiable matter sample, the previously collected aqueous phase was neutralized with 20 mL of 10% hydrochloric acid and extracted twice with 30 mL of petroleum ether by combining it with the ether extract. The combined ether extract was washed twice with distilled water and evaporated to approximately 10 mL, and then mixed with 50 mL of methanol. We added 3 mL of concentrated H_2_SO_4_ to the mixture and refluxed it for 120 min in a 95°C water bath. At the end of the reflux period, the refluxed mixture was extracted twice using 30 mL of n-hexane, with collection of the upper hexane layer. The combined hexane layer was washed thrice with equal volumes of distilled water, followed by drying at reduced pressure (below −600mBar) at 40°C to the minimum liquid volume (approximately 1–2 mL). Testing of the content of saponifiable and unsaponifiable matter in the prepared samples was performed using a GC/MS model GCMS-QP2010 SE single quadrupole Shimadzu gas chromatograph–mass spectrometer (Kyoto, Japan). The stationary phase applied for the separation was Restek Rtx-5MS—low-bleed GC-MS-fused silica capillary, equivalent to USP G27 and G36 phases (dimensions: 0.25 μm film thickness, 30 m length, 0.25 mm inner diameter) (Restek, Bellefonte, PA, USA). We launched the temperature program for the column oven at 50°C and kept it at the same temperature for 3 minutes. Subsequently, the oven temperature increased at a rate of 4.5°C/min until it reached 300°C. The oven remained at the final temperature for 10 min before returning to 50°C by the end of the run. One microliter of each sample was injected using the split mode with a split ratio equal to 1:30 and at an injection port temperature of 280°C. Mass spectrometer scan acquisition was initiated from 6 min to 63 min for each run, with an m/z scan range of 35–500. Instrument control and data acquisition were performed using the GCMS solution software (Shimadzu Corporation, Kyoto, Japan) in Total Ion Chromatogram (TIC) mode. The acquired peaks were identified through comparison of the spectra of mass fragmentation with the standard spectra of the NIST11 library. Calculation of metabolite abundance was performed on an area% basis. Chromatographic data visualization was performed using the Mass++ Software (Tanaka et al., 2014).

### 2.3 Formulation of the M-NEs

#### 2.3.1 Design and preparation of M-NE formulations using 2^2^ factorial design

Factorial design plays a crucial role in the field of pharmaceutical optimization. In this study, a 2^2^ factorial design was employed to obtain an optimized formula for M-NE utilizing Design-Expert version 11. The formulation factors were assigned as follows: type of surfactant (X1) and surfactant and co-surfactant mixture (X2). Two levels of each factor were used, as shown in Table 1. We studied the impact of these formulation factors on the responses , including droplet size (Y1) and zeta potential (ZP) (Y2).

**TABLE 1:** Formulation factors and response variables in the 2^2^ factorial design for M-NEs.

Factor and response		Level	
Number	Factor	Low (−1)	High (+1)
A: X_1_	Surfactant	Tween 20	Tween 80
B: X_2_	Surfactant and co-surfactant mixture	20%	40%
Response	Name	Goal	
Y_1_	Droplet size (nm)	Minimize	
Y_2_	Zeta potential (mV)	Maximize	

O/W marula oil nanoemulsions (M-NEs) were prepared via the high-shear homogenization-ultrasonication method (Asadinezhad et al., 2019). Four formulations were prepared with different compositions, as shown in Table 2. Accurate amounts of surfactant and co-surfactant were dissolved in double distilled water to prepare the aqueous phase. M-Oil (10 mL) was added dropwise to the aqueous phase until homogenization was completed; the total volume of oil was homogenized using a high-shear homogenizer rotating at a speed of 22,000 rpm, and it was kept under homogenization for a further 15 min (Qushawy et al., 2022). The prepared formulations of M-NE were sonicated using probe sonication for 5 min and stored at 5°C for 24 h before further investigation.

**TABLE 2:** Formulations of marula nanoemulsions (M-NEs) and their composition.

Formula no.	M-Oil % (V/V)	Type of surfactant	Surfactant % (V/V)	Co-surfactant % (V/V)	Water % (V/V)
M-NE1	20	Tween 20	10	10	60
M-NE2	20	Tween 20	20	20	40
M-NE3	20	Tween 80	10	10	60
M-NE4	20	Tween 80	20	20	40

M-oil, marula oil; M-NE, marula nanoemulsion.

#### 2.3.2 Determination of droplet size, zeta potential, and polydispersity index

We determined three parameters of the M-NE formulations (droplet size, zeta potential (ZP), and polydispersity index (PDI)) by applying the technique of dynamic light scattering using a Malvern Zetasizer (Malvern, UK). For measurement of droplet size and ZP, the samples were diluted with distilled water (1:100). We also estimated the ZP values of the formulations by determining the electrophoretic mobility of oil droplets (Rashed et al., 2022). Triplicates were used for the calculation of each parameter.

#### 2.3.3 Measurement of pH and viscosity of the M-NEs

After homogenization with water (in a 1:9 ratio), we determined the pH value of the M-NE formulations using a Jenway pH meter device (Staffordshire, UK) (Gul et al., 2022). The viscosity of the M-NE formulations was measured using the Ostwald capillary viscometer at a temperature of 25°C ± 0.5°C, and experiments were performed in triplicate (Balestrin et al., 2021). Triplicates were used for the calculation of each parameter.

#### 2.3.4 Stability testing of nanoemulsions

##### 2.3.4.1 Heating–cooling cycle (accelerated stability study)

Fifty milliliters of each M-NE formulation were transferred to a glass bottle with good sealing with the cap (n = 3) and then subjected to accelerated conditions (six cycles: 4°C and then 45°C). The physical stability of the formulations was evaluated *via* the percentage creaming (Pengon et al., 2018). We calculated the creaming index (% CI) as follows:

$$\text{CI }\%=\left[\text{CC}/\text{CT}\right]x\;100,$$

CC=the height of the creamy layer,

CT=total height of nanoemulsion layer.

#### 2.3.5 Selection of the optimized M-NE

We used version 11 of the Design-Expert software to obtain an optimized formulation of M-NEs. The best formulation of the M-NE was selected according to the lowest droplet size and highest absolute ZP.

#### 2.3.6 Transmission electron microscopy (TEM) of the optimized formulation

M-NE4 was the best formulation in terms of character and was tested for surface morphology using TEM (JTEM 1010, JEOL, Tokyo, Japan). We added 1 droplet of diluted M-NE4 to a grid coated with copper and left it until dry. Subsequently, we stained the sample by adding uranyl acetate and photographed it via TEM (Harun et al., 2018).

### 2.4 Testing of anti-inflammatory activity against rotenone-induced toxicity in mice

#### 2.4.1 Mouse environment and ethical standards

Twenty adult male Swiss albino mice (22–28 g) were purchased from the Abu Rawash Company [Giza, Egypt]. Mice were distributed equally and randomly into four experimental groups in clean polyethylene cages with free access to food and water. We housed the mice in ordinary light–dark cycles, under a maximum temperature of 30°C.

The procedures conducted as part of this work were approved by the Ethical Committee [#202203RA3] at the Faculty of Pharmacy of Suez Canal University and the Faculty of Pharmacy of Sinai University [SU-SREC-5-05-2023] and were compliant with the NIH guidelines for the use of animals. A qualified technician was responsible for the animals and for providing the feeding materials. The authors were keen to take every effort to minimize animal suffering during the experimentation and implementation of behavioral tests. Animal experiments also complied with the regulations of the European Animal Research Association and the ARRIVE guidelines.

#### 2.4.2 Experimental design and grouping of mice

After acclimatization for 10 days, animals were allocated into four groups, *n* = 5. We purchased rotenone (Sigma-Aldrich, MO, USA) and solubilized it in sunflower oil.

• The vehicle group (vehicle control) received nine subcutaneous [s.c.] injections of sunflower oil (10 mL/kg) every other day.
• The PD control group received 9 injections of rotenone [1 mg/kg, s.c.] every other day. This schedule of injecting rotenone has displayed minimal lethality when applied for induction of the PD model in mice.
• PD + crude M-Oil (M-oil) group mice received rotenone [1 mg/kg, s.c.] every other day and oral gavage of 0.2 mL of M-Oil per mouse.
• The PD + M-NE4 group received rotenone [1 mg/kg, s.c.] every other day and oral gavage of 0.2 mL of M-NE4 per mouse.

Oils were given daily at 11:00 a.m. from day 1 to day 17.

#### 2.4.3 Testing of locomotor dysfunction

##### 2.4.3.1 Open field test

A plexiglass arena with 30-cm-high walls and a 60 × 60 cm^2^ floor was used to evaluate non-forced exploring ambulation in the model mice (Alzahrani et al., 2018). Rectangular units were highlighted in an 8 × 8 cm pattern on the floor of the arena. We cleaned the apparatus between trials using alcohol. Mice were positioned individually in the center and observed for 5-min intervals; locomotor parameters were registered blindly. We recorded horizontal movement (the number of squares crossed completely by the mouse), number of stops, and number of rears. Furthermore, we calculated an activity index by dividing the number of squares crossed by the number of stops; this represents the length of a locomoting interval. Mice were not given any training beforehand to encourage them to investigate the arena.

##### 2.4.3.2 The cylinder test

Each mouse was set inside a transparent cylinder. Spontaneous activity was quantified for an interval of 3 min. Along with a count of steps performed, the number of rears was also counted. If a mouse moved both forelimbs or both hindlimbs on the ground, the forelimb and hindlimb movements were counted (Fleming et al., 2004). Rearing was defined as the rat making a full vertical movement with neither of its forelimbs touching the bench (Teema et al., 2016).

#### 2.4.4 Animal euthanasia and sample collection

Mice were euthanized *via* cervical dislocation. After that, the skull was broken to dissect the brain. One hemisphere of each brain was rapidly frozen at −80°C, while the second hemisphere was transferred to a tube containing 4% paraformaldehyde.

#### 2.4.5 Polymerase chain reaction

We measured mRNA expression of the target proteins. We extracted total RNA from striatal specimens using an SV Total RNA Isolation System from Promega (WI, USA). We estimated the RNA concentrations using a NanoDrop ND-1000 spectrophotometer. We transformed the Total RNA into cDNA with the aid of a SuperScript III First-Strand Synthesis System (#K1621, Fermentas, MA, USA). Quantification of gene expression was performed using SYBR Green Master Mix (Applied Biosystems) in a 20-μL total amplification reaction. Mouse-specific primers were used, as specified in Table 3. We determined the primer specificity using the Primer-BLAST program (NCBI/primer-BLAST) [https://www.ncbi.nlm.nih. gov/tools/primer-blast/]. We performed the PCR assays using the Applied Biosystems software package, version 3.1 (StepOne^TM^). For normalization, β-actin was employed as a housekeeping gene. We calculated the relative expression of gene mRNA, applying the comparative CT method. We normalized the values to β-actin and expressed the values as fold-change over background levels detected in the vehicle control group (Livak and Schmittgen, 2001).

**TABLE 3:** Primer sequences of genes measured in the study.

Gene	Forward	Reverse	RefSeq
IL-1β	GCC​CAT​CCT​CTG​TGA​CTC​AT	AGG​CCA​CAG​GTA​TTT​TGT​CG	NM_008361.4
TNF-α	AGA​ACT​CCA​GGC​GGT​GTC​TGT	CCT​TGT​CCC​TTG​AAG​AGA​ACC	NM_001278601.1
β-actin	CCGCGGGAGACAAGCTT	GGA​ATG​GAA​GAA​GGG​CTT​GAT​C	NM_007393.5

#### 2.4.6 ELISA assays for measurement of striatal dopamine and inflammatory cytokines

Samples from the frozen striata were homogenized in RIPA buffer and centrifuged at 2000 rpm in a cooling centrifuge, and the clear homogenates were collected. Enzyme-linked immunoassay (ELISA) kits were used for analysis of dopamine (Cat. Number: MBS732020, MyBioSource), TNF-α (Cat. Number: MBS2500421, MyBioSource), and IL-1β (Cat. Number: E-EL-M0037, Elabscience).

#### 2.4.7 Spectrophotometric assays for measurement of striatal malondialdehyde and reduced glutathione

Samples from the frozen striata were homogenized in PBS and centrifuged at 2000 rpm in a cooling centrifuge. Clear homogenates were used to measure malondialdehyde (MDA), a main product of lipid peroxidation in biological samples. The thiobarbituric acid method (Ohkawa et al., 1979) was used for the assay (Biodiagnostics kits, Cairo, Egypt). The assay procedures rely on the reaction between MDA and thiobarbituric acid at a temperature of 95°C, producing a colored compound; the intensity of the color of this compound was estimated at 534 nm using a UV–VIS spectrophotometer. We used a GSH assay kit (Biodiagnostics) to measure the GSH level in the striatal homogenates. The assay procedures included the reduction of 5,5′ dithiobis (2-nitrobenzoic acid) (DTNB) by GSH and production of a yellow compound. We measured the absorbance of the samples at 405 nm (Beutler et al., 1963).

#### 2.4.8 Western blot analysis

Extraction of total protein from tissues was performed using ice-cold RIPA lysis and extraction buffer (Thermo Fisher, USA) supplemented with Halt™ Phosphatase Inhibitor Cocktail (Thermo Fisher, USA). Following the manufacturer’s instructions, the Bio-Rad Protein Assay Kit (Bio-Rad, USA) was employed to measure the protein concentration. The proteins were denatured through boiling in 4x Laemmli sample buffer (Bio-Rad, USA). Equal quantities of proteins and a pre-stained protein molecular weight marker (Catalog no. 161-0305, Bio-Rad, USA) were separated using SDS-PAGE (10%). Using electroplating equipment, the samples were transferred to polyvinylidene fluoride (PVDF) membranes and then incubated in 5% skim milk (a blocking agent) for one hour at 37°C. Primary antibodies against tyrosine hydroxylase (1:1000, PA1-4679, Thermo Fisher, USA), alpha-synuclein (1:500, PA1-18264, Thermo Fisher, USA), and β-actin (1:2000, PA1-183, Thermo Fisher, USA) were incubated overnight at 4°C on the membranes. After incubation with the secondary antibody, enhanced chemiluminescent substrate (ECL, Amersham BioSciences, UK) was used to detect bound proteins according to the manufacturer’s protocol. Densitometric analysis was performed to compare the band intensity of the target protein to the control protein β-actin *via* protein normalization after scanning of the membranes using a ChemiDoc MP Imaging System (Bio-Rad).

### 2.5 Histopathology

The formalin-fixed sections from the experimental groups were processed and embedded in Paraplast tissue embedding media. Subsequently, we cut 4-μm sagittal brain sections to demonstrate the substantia nigra in specimens. Next, brain sections were dewaxed and rehydrated before staining, followed by toluidine blue (TB) staining for demonstration and quantitative analysis of apparent intact neurons. An experienced pathologist blinded to the experimental condition examined the stained tissue sections using the Full HD light microscopic imaging system (Leica Microsystems GmbH, Wetzlar, Germany) (Culling, 2013). Photomicrographs were captured and analyzed using the Leica application module for histological analysis, which was attached to the Full HD microscopic imaging system (Leica Microsystems GmbH, Germany).

### 2.6 Data handling and statistical analysis

The SPSS program and GraphPad Prism software were utilized to process statistical data. We present the data in the form mean ± SD. We used the Shapiro–Wilk test to check for normality of distribution, with a threshold of *p* < 0.05, and subsequently applied one-way analysis of variance (ANOVA). Pair-wise comparisons were carried out using the Bonferroni test.

## 3 Results and discussion

### 3.1 M-Oil characterization

**FIGURE 1:**
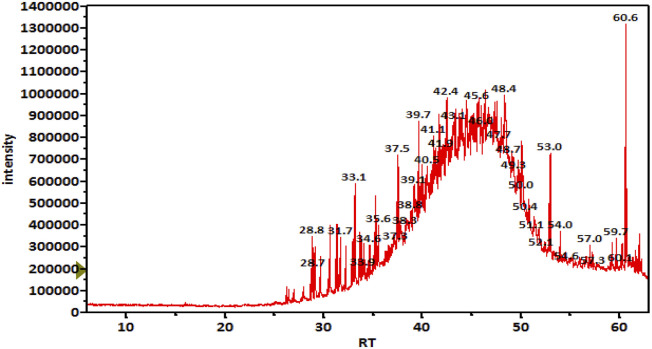
TIC chromatogram of unsaponifiable matter of an M-Oil working sample.

GC-MS analysis (Figure 1) and MS library spectral matching of the unsaponifiable matter content resulted in the tentative identification of several sterol metabolites (Table 4), such as (metabolite, area% of total unsaponifiable matter) γ-sitosterol, 9.14%; 9,19-cyclolanost-24-en-3-β-ol, 1.54%; stigmasterol, 1.07%; obtusifoliol, 1.00%; and ergost-5-en-3-β-ol, 0.88%. In addition, squalene, phytol, and alkane wax matter of plant origin were observed (e.g., octacosane, eicosane, 2-methylhexacosane, heneicosane, tetratetracontane, and tetracontane).

**TABLE 4:** Important metabolites detected in unsaponifiable matter of an M-Oil sample, ordered by retention time.

Retention time (min)	Area%	Compound name	m/z
52.967	2.95	Squalene	69.05
59.195	0.88	Ergost-5-en-3-β-ol	43.05
59.680	1.07	Stigmasterol	55.05
60.251	1.00	Obtusifoliol	55.05
60.652	9.14	γ-Sitosterol	43.05
61.992	1.54	9,19-Cyclolanost-24-en-3-β-ol	69.05

**FIGURE 2:**
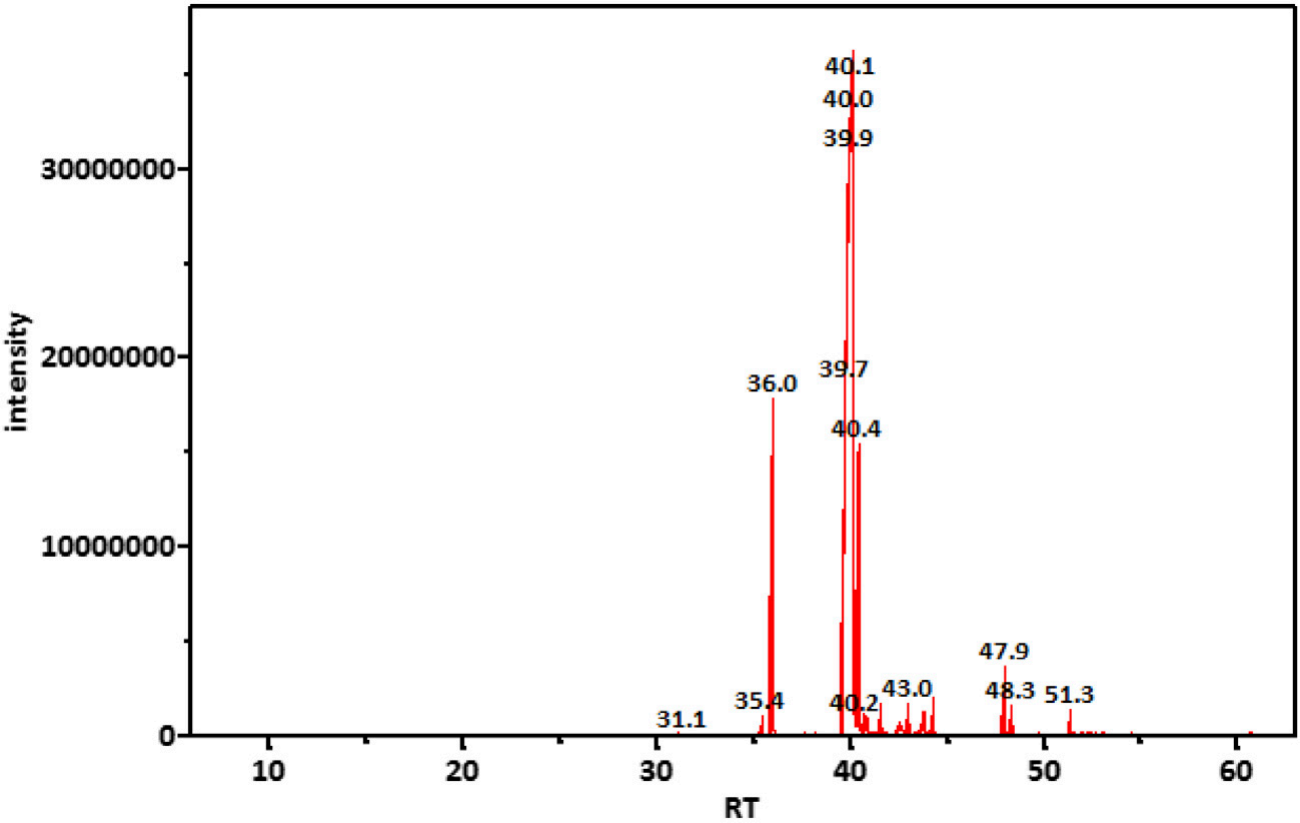
TIC chromatogram of saponifiable matter of the tested M-Oil sample analyzed as FAME.

On the other hand, TIC of the saponifiable matter analyzed as fatty acid methyl esters (FAME) (Figure 2) revealed that the major constituents of the tested oil sample were as follows (metabolite name, area% from total chromatogram): methyl 9-cis-11-trans-linoleate, 50%; methyl oleate, 31%; methyl palmitate, 8.3%; methyl stearate; 5.4%; and docosanoic acid methyl ester, 1% (Table 5).

**TABLE 5:** Major constituents of the saponifiable matter of marula samples calculated as area% of FAME.

Retention time (min)	Area%	Compound name	m/z
35.99	8.28	Methyl palmitate	74.00
39.97	49.67	Methyl 9-*cis*-11-*trans*-linoleate	67.05
40.13	30.74	Methyl oleate	55.05
40.40	5.36	Methyl stearate	74.05
47.95	1.02	Docosanoic acid, methyl ester	74.00

Data are ordered according to retention time.

### 3.2 Droplet size, ZP, and PDI of M-NEs

We formulated four M-NE preparations via the high-shear homogenization technique and determined their droplet size, PDI, ZP, pH, viscosity, and CI %.

**FIGURE 3:**
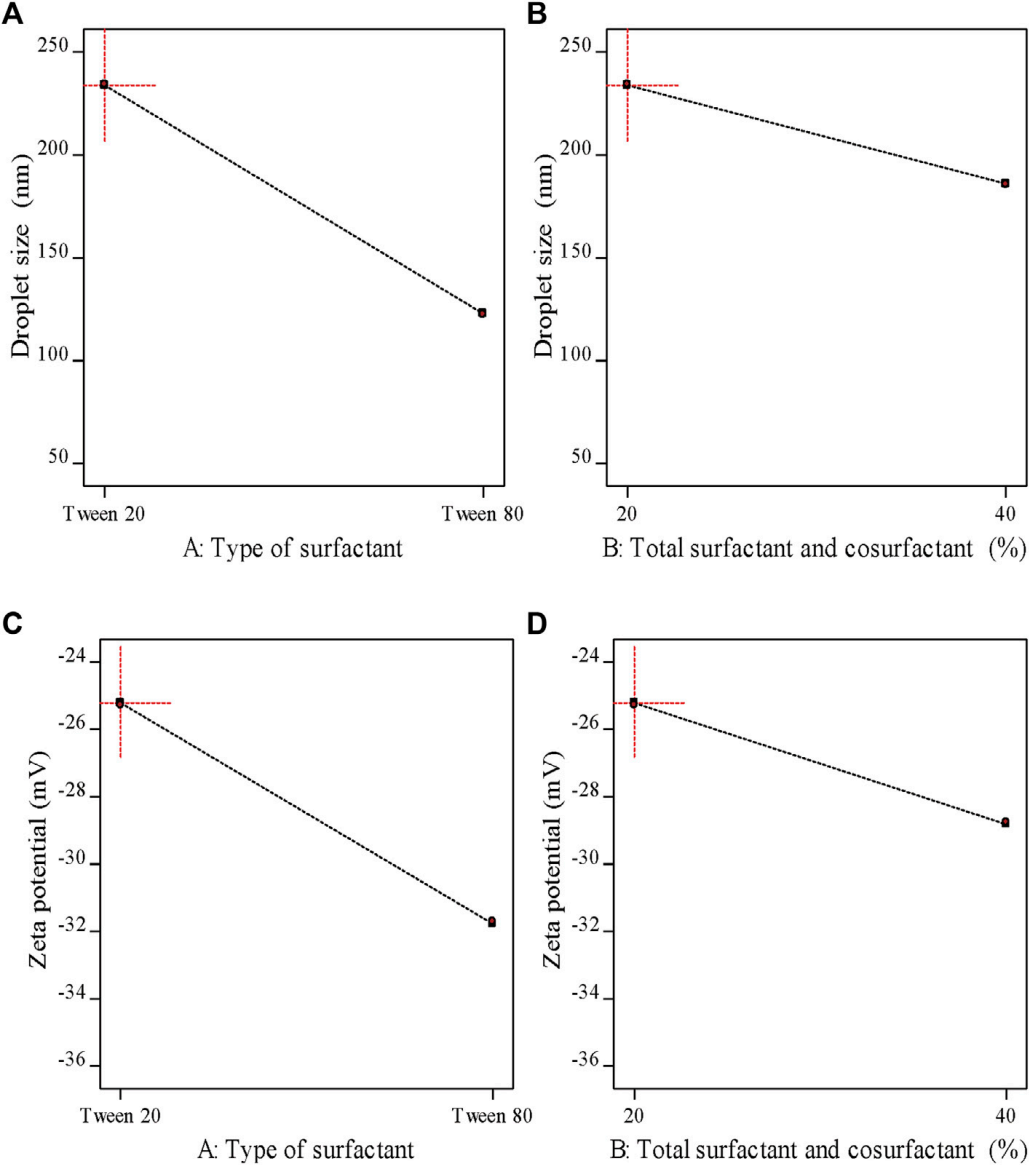
Main effects plots demonstrating the impact of each formulation factor on different response variables.

As demonstrated in Table 6 the prepared M-NEs exhibited small droplet sizes ranging from 75.36 ± 0.95 nm for M-NE4 to 234.28 ± 2.91 for M-NE1. It was found that the droplet size was smaller in formulations that were prepared by Tween 80 (HLB = 14.9) than in formulations prepared by Tween 20 (HLB = 16.7) (Figure 3). These variable results may be attributed to variation in the HLB values of the surfactant used; the lower the HLB value, the higher the lipophilicity, which results in a decrease in the surface free energy and hence a smaller droplet size. Liu et al. published similar results; they developed a lemon oil nanoemulsion and documented increased droplet size as the HLB value of the surfactant increased (Liu et al., 2022). Furthermore, we observed decreased droplet size as the amount of surfactant and co-surfactant mixture increased. These results can be explained by the reduced interfacial tension between the oil phase and aqueous phase. Additionally, the PDI value provides an indication of the homogeneity of the droplet size (Chinnaiyan et al., 2022). It was found that all M-NE formulations had a low PDI of less than 0.5, which indicates homogeneity of droplet size (Table 6).

**TABLE 6:** Evaluation of droplet size, PDI, ZP, pH, viscosity, and CI% in marula nanoemulsions.

Formula number	Droplet size (nm)	PDI	ZP (mV)	pH	Viscosity (cp)	CI %
M-NE1	234.28 ± 2.91	0.385 ± 0.02	−25.28 ± 1.24	6.28 ± 0.01	3.45 ± 1.02	10.40 ± 0.60
M-NE2	185.63 ± 1.75	0.267 ± 0.01	−28.76 ± 0.74	6.92 ± 0.03	6.98 ± 1.28	8.01 ± 0.60
M-NE3	122.48 ± 1.29	0.319 ± 0.01	−31.71 ± 1.34	6.22 ± 0.02	10.18 ± 1.65	5.50 ± 0.33
M-NE4	75.36 ± 0.95	0.377 ± 0.02	−35.44 ± 1.04	6.53 ± 0.03	15.64 ± 1.74	3.72 ± 0.35

Data are presented in the form mean ± SD; M-NE, marula nanoemulsion; CI, creaming index; *n* = 3.

ZP is the magnitude of the charge that is present at the border between the two phases of a nanoemulsion. As shown in Table 6 , all M-NE formulations were found to exhibit high negative ZP. The negative charge may be attributed to M-Oil, which is composed of high proportions of oleic acid and linoleic acid (Kamanula et al., 2022; Chivandi et al., 2023). The negative value was increased by using Tween 80 instead of Tween 20 and by increasing the amount of surfactant and co-surfactant mixture (Figure 3). These findings may be explained by the smaller droplet size obtained using Tween 80 and larger amounts of surfactant and co-surfactant mixture, which led to larger surface area and hence the increased surface charge. ANOVA of the droplet size and ZP results indicated that both X1 and X2 exerted significant effects on both of these variables (*p* < 0.05, Supplementary Table S1).

### 3.3 Characterization of M-NEs

As shown in Table 6, the pH values of the formulations ranged from 6.22 ± 0.02 to 6.92 ± 0.03, whereas the viscosity of the M-NEs showed a lower value of 3.45 ± 1.02 cP and a maximal value of 15.64 ± 1.74 cP. The viscosity of formulations that were prepared using Tween 80 was higher than that of formulations prepared using Tween 20. We believe that these results can be accounted for by the difference in viscosity between Tween 80 and Tween 20.

The CI% of M-NEs provides an indication of the physical stability of the formulations. All preparations showed low CI% values, ranging from 3.72% ± 0.35% for M-NE4 to 10.40% ± 0.60% for M-NE1. As shown in Table 6, the CI% value had an inverse relationship with viscosity: the higher the viscosity, the lower the CI% value.

### 3.4 Selection of the best formulation

We used a 2^2^ full factorial design for optimization of the formulation factors that resulted in an optimal M-NE with the target responses using the Design-Expert software, version 11. The aim of optimization of the prepared M-NE was to obtain an optimized formula with minimal droplet size (Y1) and maximum ZP (Y2). As shown in Supplementary Figure S1, the optimized formulation was M-NE4, which was prepared with the high level of X1 (+1, Tween 80) and the high level of X2 (+1, 40%). We found that the predicted value was close to the actual value and the desirability was 0.997, indicating the application of a valid factorial design (Hosny et al., 2021).

### 3.5 Surface morphology of formula M-NE4

**FIGURE 4:**
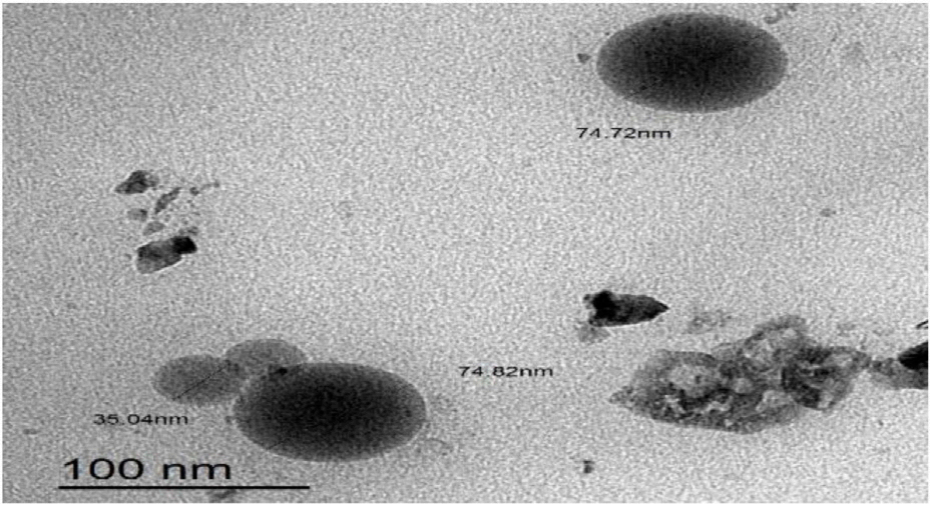
TEM image of the best marula oil nanoemulsion formulation (M-NE4).

As illustrated in Figure 4, M-NE4 appeared spherical, with a smooth surface with no aggregates. Mahdi et al. documented similar data with a cranberry seed oil nanoemulsion and found that TEM images showed nanodroplets with aggregation and uneven spherical shapes (Mahdi et al., 2022).

### 3.6 *In vivo* anti-parkinsonian activity

#### 3.6.1 Locomotor function

##### 3.6.1.1 The open field test

**FIGURE 5:**
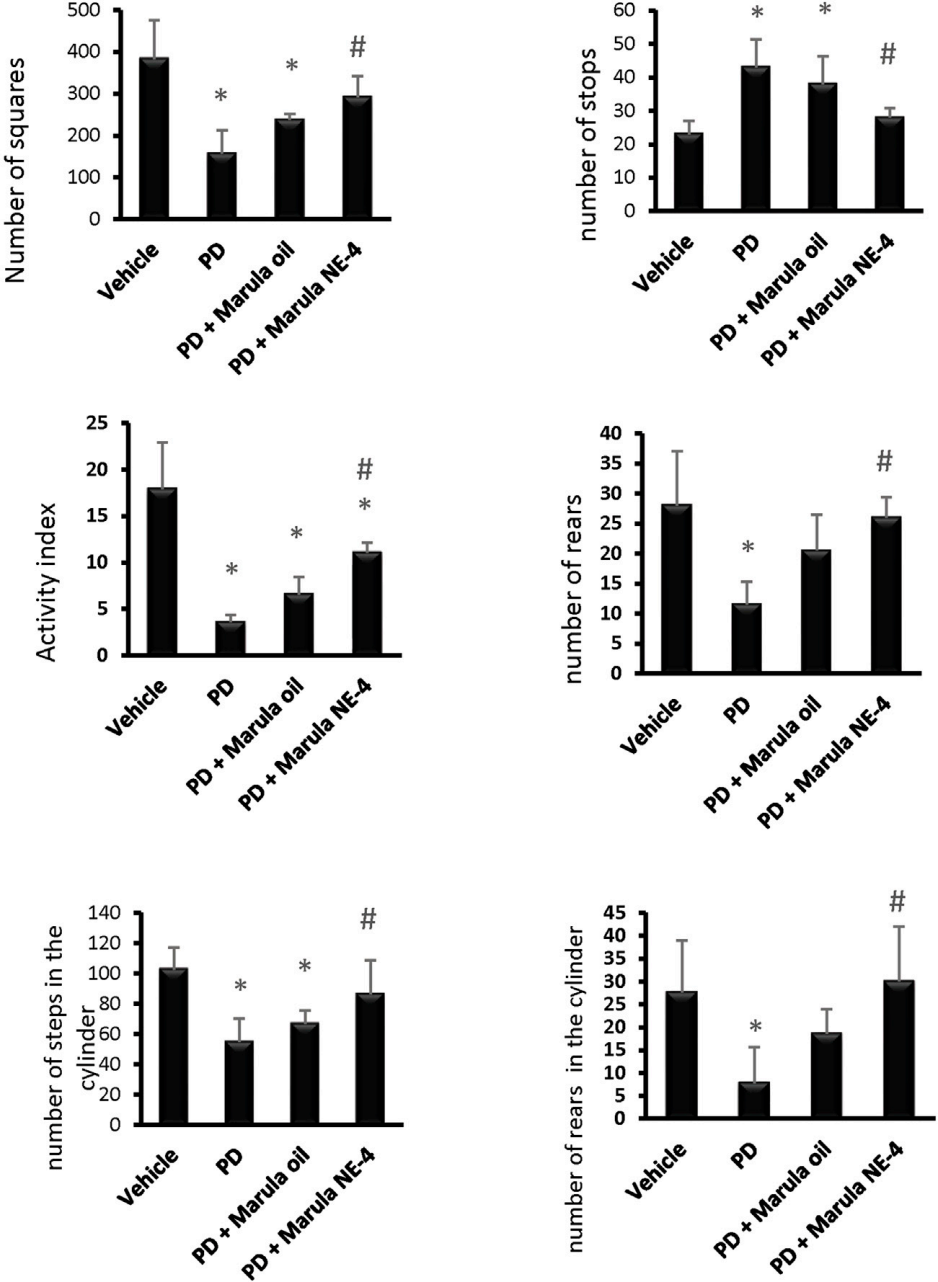
Effects of marula oil and marula NE4 on the locomotor activity of parkinsonian mice. Data plotted represent the mean ± SD for parameters measured in the open field test and cylinder test. One-way ANOVA and Bonferroni tests were conducted, with a threshold of *p* < 0.05 (n = 5). Symbols indicate significant differences compared to: (*) the vehicle group, (#) the PD control group, or ($) the PD + marula oil group.

A one-way ANOVA indicated a difference between the study groups in the number of squares crossed within 5 min (F(3,16) = 11.33), number of stops (F(3,16) = 10.19), activity index (F(3,16) = 25.65), and frequency of rearing (F(3,16) = 8.05), as shown in Figures 5A–5D. *Post hoc* analysis demonstrated that the PD control group showed fewer crossed squares, a lower activity index, and a lower frequency of rears than the vehicle group. In contrast, the PD + M-NE4 group displayed significant increases in these parameters compared to the PD control group; however, the PD + crude M-Oil group did not display meaningful differences compared to the PD control group.

##### 3.6.1.2. Cylinder test

Similarly, an ANOVA indicated differences in the parameters measured in the cylinder test, both stepping (F(3,16) = 8.78) and rears (F(3,16) = 5.78), as shown in Figures 5E, F. The PD control group showed fewer steps and a lower frequency of rears than the vehicle group (Figures 5E, F). In contrast, the PD + M-NE4 group exhibited significant increases in these parameters compared to the PD control group; however, the PD + crude M-Oil group did not display significant differences compared to the PD control group. Amelioration of motor dysfunction was observed in the groups that received M-NE4 in comparison to mice that received the crude M-Oil. Most of the parameters measured in the open field test were ameliorated in a significant way by M-NE4. Many plant oils have been studied thoroughly to investigate their potential ameliorative effects on locomotor ability in parkinsonism. M-Oil has not previously been addressed in the treatment of parkinsonism despite its suggested anti-inflammatory and antioxidant effects (Mariod and Abdelwahab, 2012). Amelioration of experimental PD is accurately assessed by motor improvement, as used in many studies addressing the potential ameliorative effects of certain novel molecules (Zaitone et al., 2019).

**FIGURE 6:**
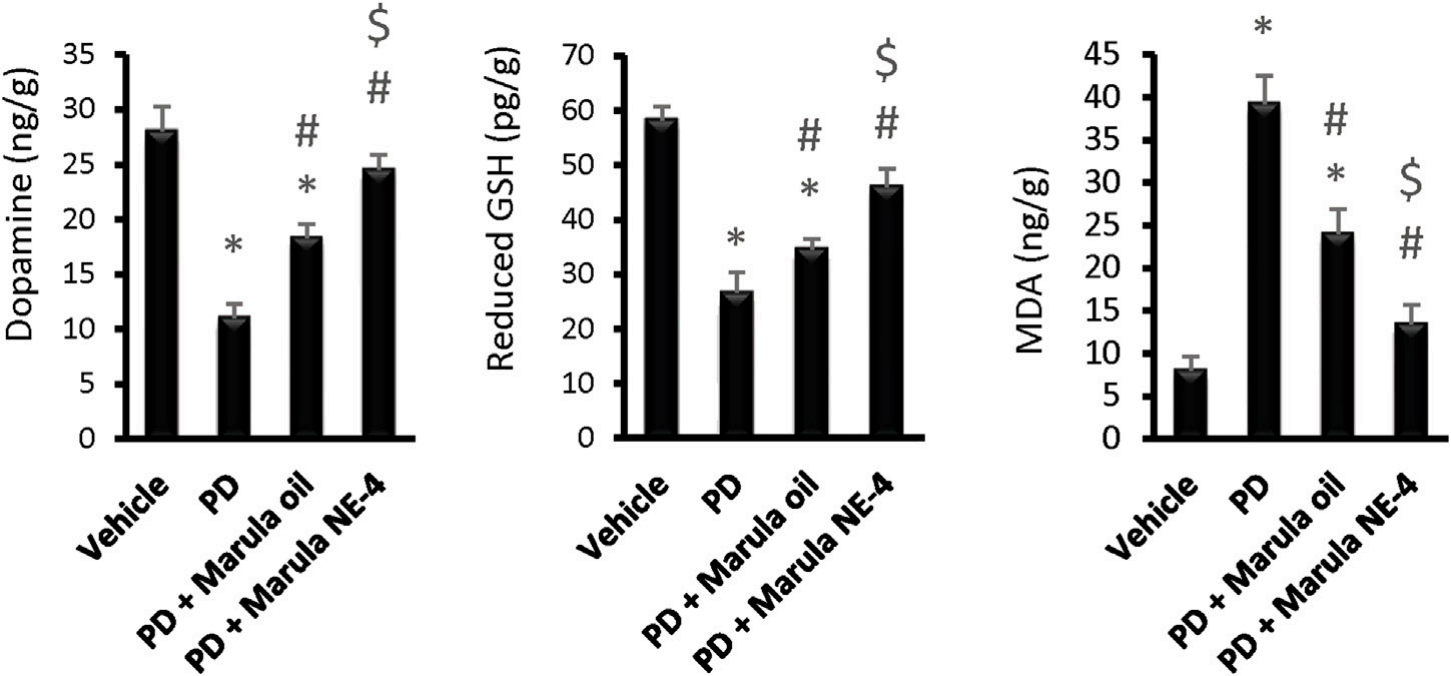
Effects of marula oil and marula-NE4 on striatal dopamine, GSH, and malondialdehyde levels. Data plotted represent the mean ± SD; one-way ANOVA and Bonferroni tests were conducted, with a threshold of *p* < 0.05. Symbols indicate significant differences compared to: (*) the vehicle group (*n* = 5), (#) the PD control group, or ($) the PD + marula oil group.

Application of the ANOVA indicated differences in the parameters measured in the striatal samples, i.e., dopamine (F(3,16) = 111.19), GSH (F(3,16) = 105.89, and MDA (F(3,16) = 139.98), as shown in Figures 6A–C. The PD control group showed significant reductions in striatal dopamine and GSH levels, but groups treated with M-Oil or M-NE4 showed amelioration of these changes. Importantly, treatment with M-NE4 produced better responses than treatment with crude M-Oil (Figures 6A–C). Parkinsonism presents initially in 89% of subjects with rigidity and akinesia, and this is found to be highly correlated with dopamine levels in the basal ganglia (Moustafa et al., 2016). This rigidity can be assessed easily through the activity index, as rigidity causes slowness of movement (hypokinesia) and can lead ultimately to akinesia (Kann et al., 2020). This was evident in PD groups, as the lowest values for the activity index were recorded in these mice; amelioration was proven in mice treated with the M-NE.

Moreover, the cylinder test also showed significant improvement in groups receiving M-NE. In addition, the types of movement (horizontal and vertical) were improved. This proves that rigidity is ameliorated, permitting free movement of limbs. As mentioned previously, improved rigidity is tightly correlated with basal ganglia dopamine content.

**FIGURE 7:**
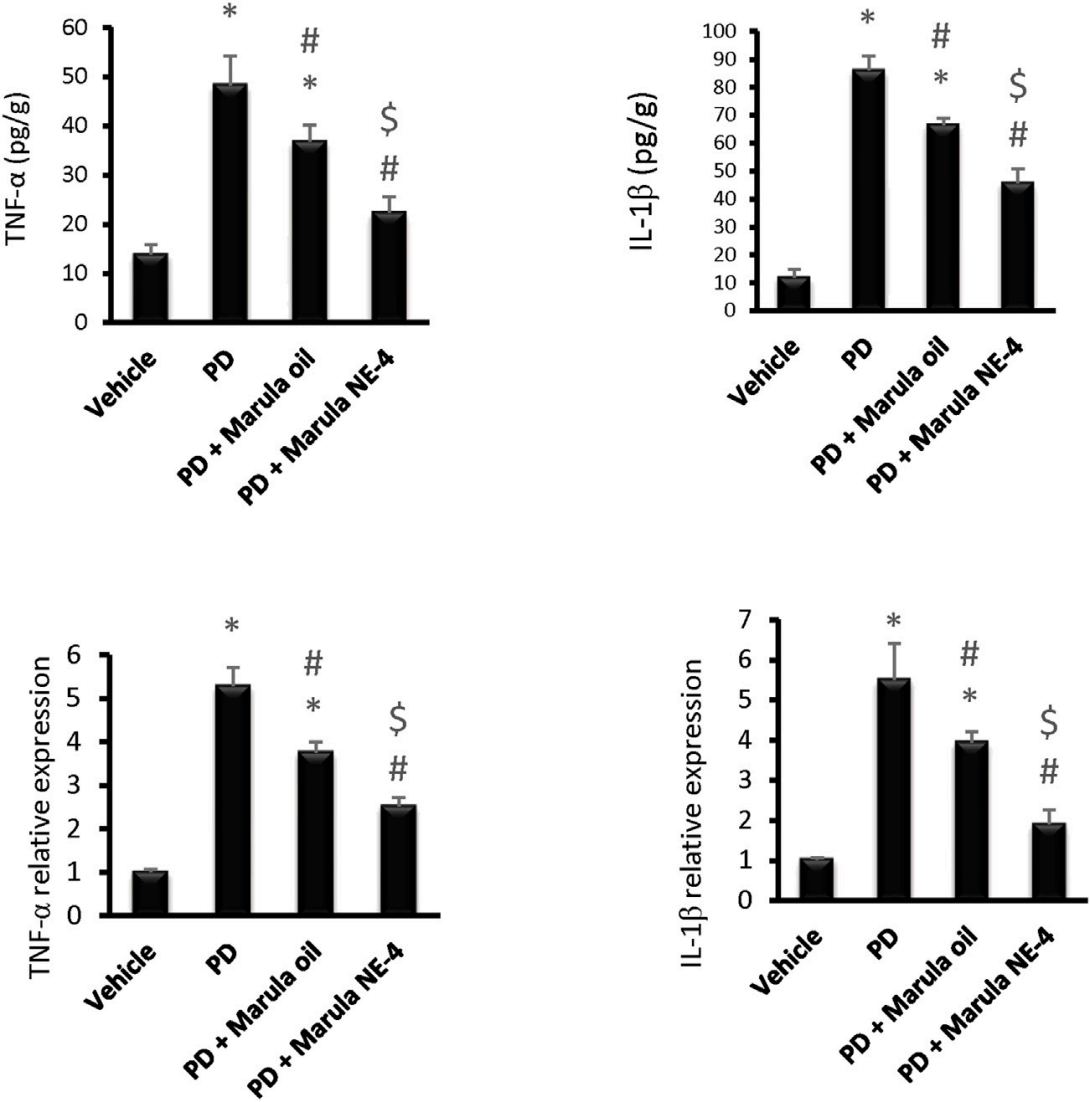
Effects of marula oil and marula-NE4 on protein levels and mRNA expression of inflammatory cytokine. Data plotted represent the mean ± SD; one-way ANOVA and Bonferroni tests were conducted, with a threshold of *p* < 0.05 (*n* = 5). Symbols indicate significant differences compared to: (*) the vehicle group, (#) the PD control group, or ($) the PD + marula oil group.

Application of the ANOVA test indicated differences in the parameters measured in the striatal samples, i.e., TNF-α protein (F(3,16) = 77.28), IL-1β protein F(3,16) = 293.5, TNF-α expression (F(3,16) = 234.7), and IL-1β expression (F(3,16) = 81.14), as shown in Figures 7A–D. The PD control group showed significant elevation in the protein levels and in expression of the inflammatory markers measured.

**FIGURE 8:**
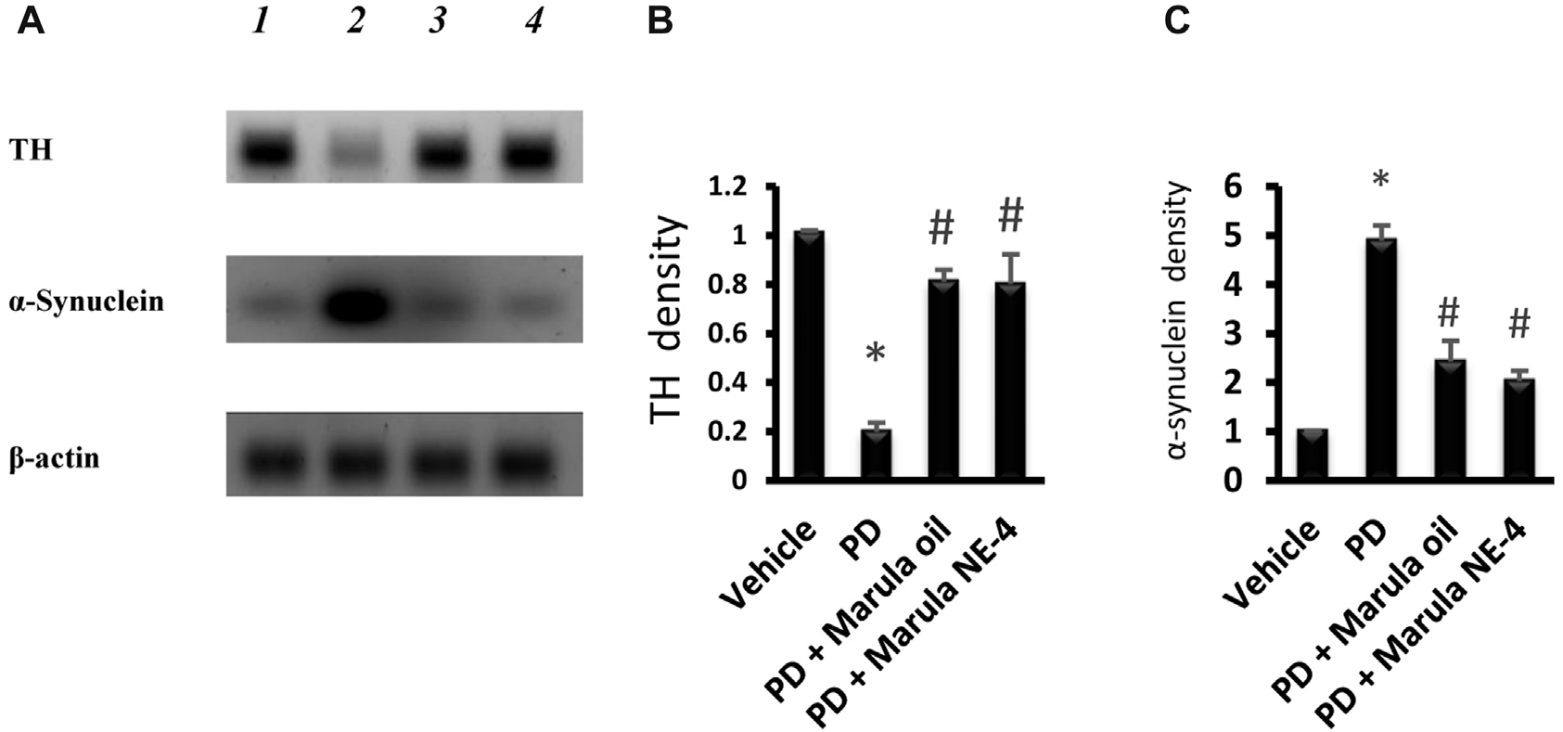
Western blot analysis for tyrosine hydroxylase and α-synuclein proteins. **(A)** Western blot gels for the selected markers. **(B, C)** Bar charts indicating the density of proteins. Data plotted represent the mean ± SD; one-way ANOVA and Bonferroni tests were applied with a threshold of *p* < 0.05 (*n* = 5). Symbols indicate significant differences compared to: (*) the vehicle group, or (#) the PD control group.

Figure 8A presents the protein bands cropped from the original WB gels. Western blotting indicated low protein levels for TH but greater α-synuclein in the PD control group compared to the vehicle (Figures 8B–C). However, the PD + M-Oil and PD + M-NE4 groups showed significant increases in TH protein and reductions in α-synuclein when compared to the PD control group.

As illustrated in Supplementary Figure S2, substantia nigra neurons in the vehicle group showed intact neurons with visible nuclei (Panel A), whereas the PD group showed low numbers of viable neurons (Panel B). Panel C illustrates the moderate number of viable neurons observed in the PD + M-Oil group, while Panel D illustrates a high number of viable neurons. Panel E shows the statistical analysis of differences between the study groups; the number of intact neurons in the PD group was significantly lower than the number detected in the vehicle group. Importantly, mice in the PD + M-NE4 group showed a significant increase in the number of intact neurons compared to PD mice.

Previous studies have indicated nigral neurodegeneration and inflammatory burden in the striata of rotenone-treated rodents. Teama et al. documented greater striatal cyclooxygenase-2 and vascular endothelial growth factor and lower TH immunostaining *versus* vehicle-treated rats, and reported that treatment with NSAIDs reduced the inflammatory burden, alleviated the motor symptoms accompanying rotenone-induced parkinsonism, and reduced neuronal degeneration and pyknosis in the substantia nigra (Teema et al., 2016). In agreement with these findings, RT-PCR analysis was performed in one previous study, with the results emphasizing upregulated genes encoding CD11b (a microglia surface antigen), COX-2, inducible nitric oxide synthase (iNOS), and NFκB (Zaitone et al., 2019). Similarly, Alzahrani et al. found significant increases in the expression of striatal COX2 and iNOS, along with CD11b (Alzahrani et al., 2018). Siracusa et al. reported increased levels of NFκB, iNOS, and NRLP3 in rotenone-treated parkinsonian mice (Siracusa et al., 2020). In accordance with our findings, a recent study has reported upregulation in HMGB1/TLR4 in rats with parkinsonism induced by rotenone (El-Sayed et al., 2023). Finally, another study in BV2 microglia cells demonstrated that rotenone stimulated the release of IL-1β and IL-6 (Liang et al., 2017).

## 4 Conclusions

There was no previously published work assessing the neuroprotective effects of M-Oil and its nanoemulsions. Accordingly, a single-dose study design was employed, as the current work was an early-stage exploratory study. This decision was supported by the compliance of the work with the 4R rules (reduce, refine, replace, and responsibility) for the use of experimental animals, but it can still be considered a limitation of the current study.

In the current PD model, M-Oil showed promising suppression of neuroinflammation induced by rotenone, justifying further research and development. We can conclude that formulating M-Oil as a nanoemulsion enhanced its physical characteristics. This finding was supported by enhanced biological activity, indicating its role as an anti-inflammatory and antioxidant agent that was able to downregulate the inflammatory burden and alleviate locomotor dysfunction in experimental PD in mice. Further studies are warranted to test M-Oil formulations in other neurological disorders and to verify its mechanism of action.

## Data Availability

The raw data supporting the conclusion of this article will be made available by the authors, without undue reservation.
