# Supplementary figures and images for: Marula oil nanoemulsion improves motor function in experimental parkinsonism via mitigation of inflammation and oxidative stress
Source: Front Pharmacol. 2023 Nov 23;14:1293306. doi: 10.3389/fphar.2023.1293306 (PMC10729903; doi:10.3389/fphar.2023.1293306)

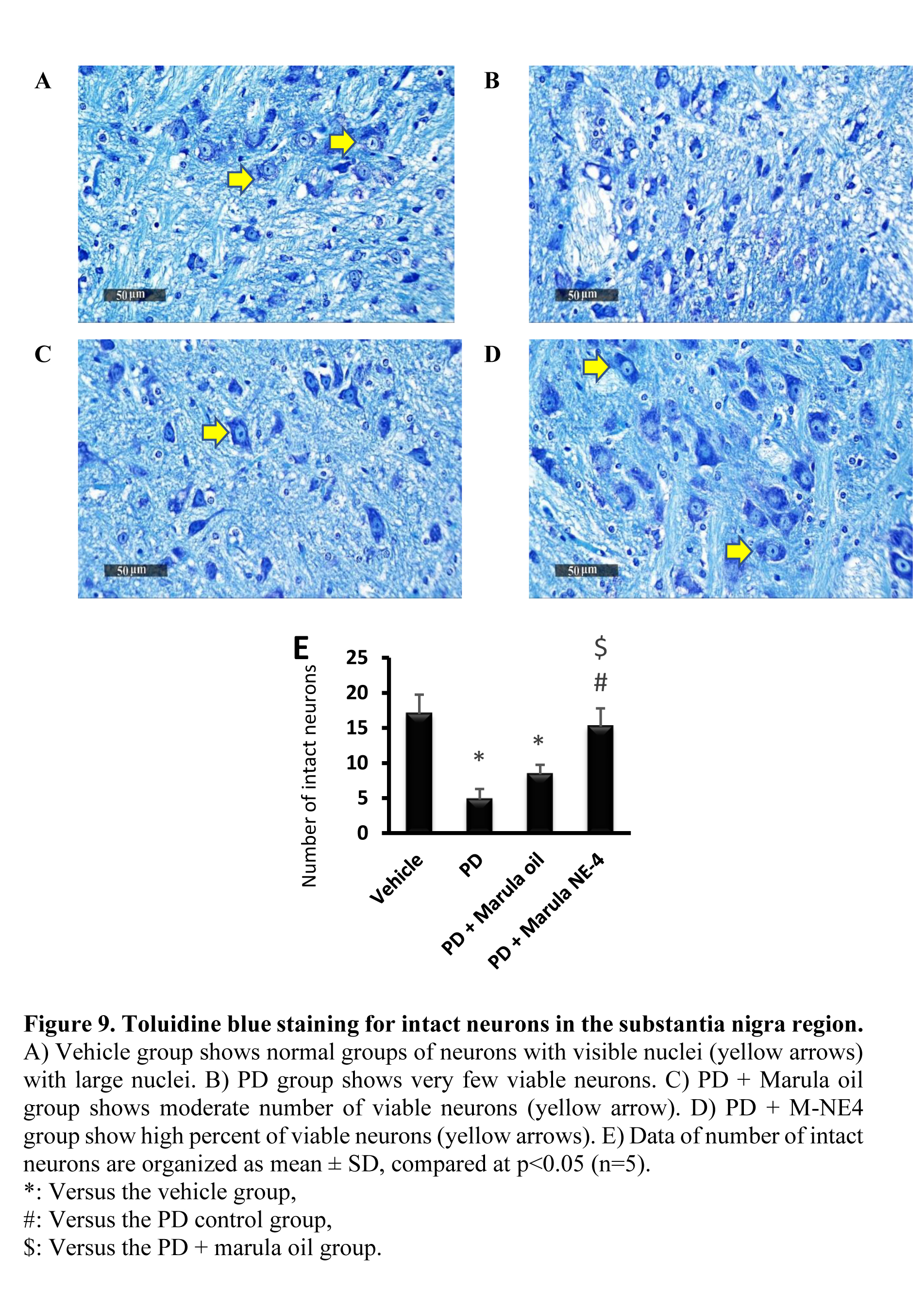

Supplement: Supplementary file 1 [file Image2.jpg]

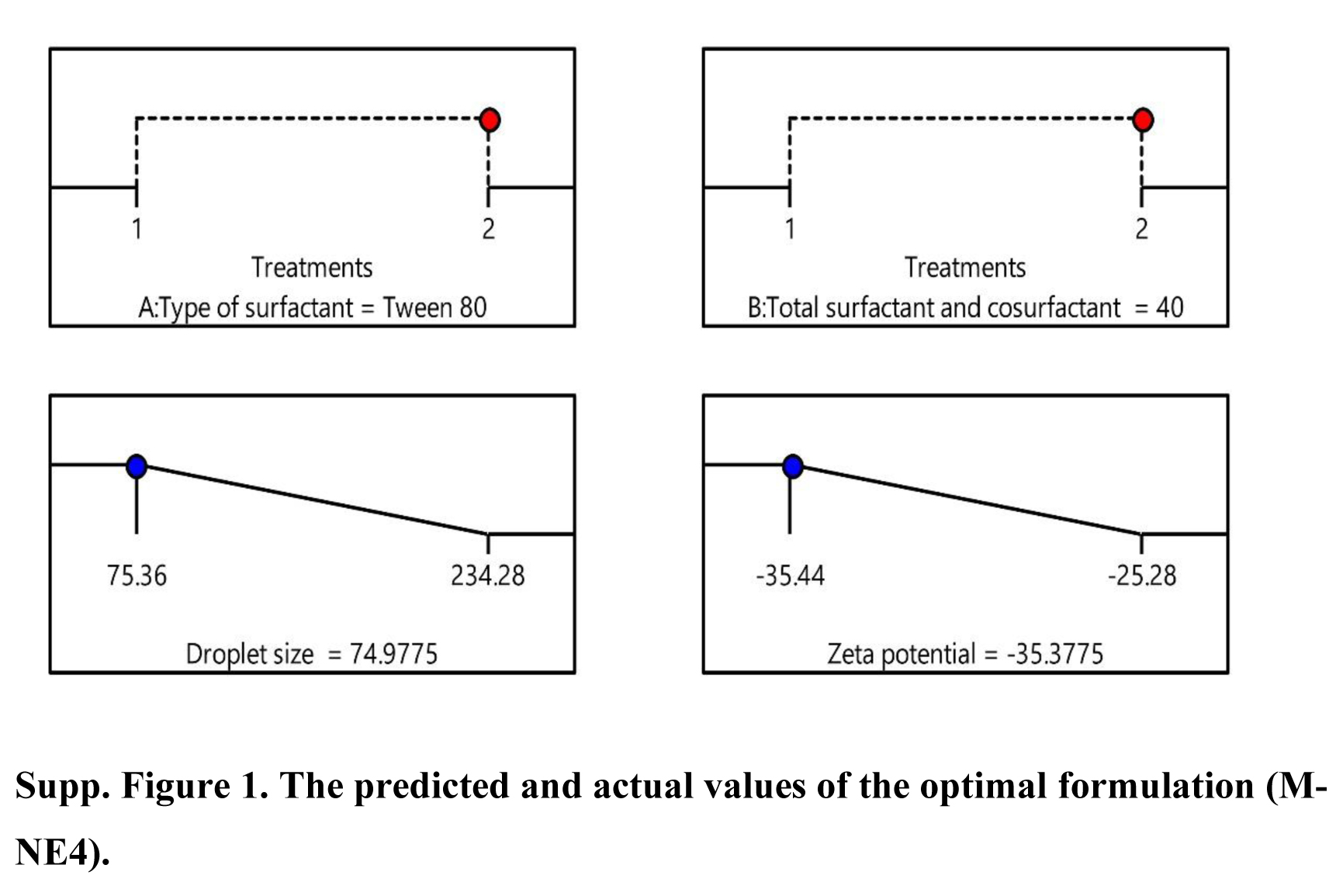

Supplement: Supplementary file 3 [file Image1.jpg]
