# Supplementary material for: Marula oil nanoemulsion improves motor function in experimental parkinsonism via mitigation of inflammation and oxidative stress
Source: Front Pharmacol. 2023 Nov 23;14:1293306. doi: 10.3389/fphar.2023.1293306 (PMC10729903; doi:10.3389/fphar.2023.1293306)
Supplement: Supplementary file 2 [file DataSheet1.pdf]

Supplementary Table 1. ANOVA analysis for (Y1), and (Y2) of the prepared M-NE.

Response 1: Droplet size					
Source	Sum of Squares	Degree of freedom	Mean Square	F value	P value
Model	14621.74	2	7310.87	12492.41	0.0063
A-Type of surfactant	12328.77	1	12328.77	21066.72	0.0044
B-Total surfactant and cosurfactant	2292.97	1	2292.97	3918.11	0.0102
Residual	0.5852	1	0.5852		
Cor Total	14622.33	3			

Response 2: ZP					
Source	Sum of Squares	Degree of freedom	Mean Square	F value	P value
Model	55.96	2	27.98	1790.85	0.0167
A-Type of surfactant	42.97	1	42.97	2749.95	0.0121
B-Total surfactant and cosurfactant	13.00	1	13.00	831.75	0.0221
Residual	0.0156	1	0.0156		
Cor Total	55.98	3			
